# Regulators of interferon-responsive microglia uncovered by Genome-wide CRISPRi screening

**DOI:** 10.1038/s44400-026-00125-4

**Published:** 2026-09-02

**Authors:** Amanda McQuade, Vincent Cele Castillo, Venus Hagan, Weiwei Liang, Thomas Ta, Reet Mishra, Olivia Teter, Layla Gomes, Bianca Gonzalez, Noam Teyssier, Kun Leng, Martin Kampmann

**Affiliations:** 1 Institute for Neurodegenerative Diseases, University of California, San Francisco, San Francisco, CA USA; 2 UC Berkeley-UCSF Graduate Program in Bioengineering, University of California, San Francisco, San Francisco, CA USA; 3 Engineering and Technology Department, City College of San Francisco, San Francisco, CA USA; 4 Biomedical Informatics Graduate Program, University of California, San Francisco, San Francisco, CA USA; 5 Medical Scientist Training Program, University of California, San Francisco, San Francisco, CA USA; 6 Biomedical Sciences Graduate Program, University of California, San Francisco, San Francisco, CA USA; 7 Department of Biochemistry and Biophysics, University of California, San Francisco, San Francisco, CA USA

**Keywords:** Computational biology and bioinformatics, Immunology, Neurology, Neuroscience

## Abstract

Microglia dynamically support brain health through the induction of specialized activation states in response to injury or disease. Activation of the interferon-responsive microglia (IRM) state has been identified across neurodevelopmental windows, age-related cognitive decline, and neurodegenerative diseases. Functionally, IRM have been linked to synaptic pruning, dead cell removal, and neuroinflammation, making this state critical to brain homeostasis. While the functional importance of this state is becoming increasingly clear, our understanding of the regulatory networks that govern IRM induction remain incomplete. To systematically identify genetic regulators of the IRM state, we conducted a genome-wide CRISPR interference screen in human iPSC-derived microglia using IFIT1 as a representative IRM marker. We identified 772 genes that modulate IRM, including canonical type I interferon signaling genes (*IFNAR2, TYK2, STAT1/2, USP18*) and newly described regulators. We uncovered a non-canonical role for the CCR4-NOT transcription complex subunit 10, *CNOT10*, in IRM activation. This work provides a comprehensive resource that can be applied to dissect the functions of interferon-responsive microglia and highlights both established and novel targets for modulating microglial interferon signaling in health and disease.

## Introduction

Microglia are dynamic immune cells that continuously monitor the brain parenchyma to maintain brain health^1,2^. In response to insult or injury, microglia rapidly trigger transcriptional activation programs to restore homeostasis. These immune responses were traditionally characterized as merely a response to pathology, however, the last decade of research has centered microglial activation as a disease-modifying pathway across many developmental and neurodegenerative conditions^3–8^.

One microglial activation state that has a clear functional impact on brain health is the Interferon-Responsive Microglia (IRM) state^9–14^. IRM are defined by high expression of interferon-stimulated genes (ISGs) and are associated with clearance of dead cells and reduced neuronal synapses. Expression of ISGs was originally characterized as a mechanism of host defense against viral particles. However, microglia have been found to strongly induce ISGs even in the absence of viral infection^10,15–17^. The nucleic acid recognition pathways that induce ISGs can be triggered by microRNAs, leakage of mitochondrial DNA, or LINE elements that leak into the cytoplasm^9,18–20^. Additionally, IRM can be triggered from paracrine signaling of IFN produced in other cell types and tissues, for example the T cells or the choroid plexus^11,15,21^.

In development, IRM play critical roles in synaptic refinement^13^. Increased levels of IRM have been detected in Autism Spectrum Disorder (ASD) patients^22^ and in in vitro experiments modeling ASD de novo mutations^23^. In normal development, IRM activation is tightly temporally regulated within specific developmental windows, and does not persist into healthy adulthood. Interestingly, in the aging brain, IRM expand again. Over half of the genes differentially expressed in young vs. aged microglia are related to Type I interferon responses^24,25^. Furthermore, in recently established brain aging clocks, nearly all brain cell types show a strong interferon response signature in the aged brain, which is hypothesized to be linked to the normal cognitive decline associated with aging^26^. Additionally, IRM show significant enrichment in neurodegenerative disease conditions including Alzheimer’s disease^10,16,27^.

Despite the identification of IRM across aging and disease datasets, it is still not fully understood if IRM represent a beneficial activation program, working to combat aging and disease phenotypes, or if IRM are maladaptive and contribute to disease progression. The impact of IRM is likely to be disease-, pathology-, and temporally-specific. Current evidence suggests that increased interferon signaling in the brain enhances microglial phagocytosis, potentially inducing aberrant synaptic pruning, which leads to dementia-like symptoms^10,14,16^. Blocking interferon responses with transgenic mice lacking the type I interferon receptor (IFNAR1), anti-IFNAR antibodies, or cGAS-STING inhibitors that reduce IRM has shown efficacy in slowing cognitive decline in normal aging contexts as well as in Alzheimer’s disease models, driven by either amyloid or tau, and in Parkinson’s disease^9–12,28^. However, IFNβ and IRM are not always detrimental. For example, IFNβ has been used to treat patients with multiple sclerosis^29,30^. In Experimental Autoimmune Encephalomyelitis (EAE) models of Multiple Sclerosis, activation of microglial interferon responses, specifically through ganciclovir treatment, proved therapeutic. Similarly, IFNβ stimulation promoted beneficial microglia functions, including phagocytosis of α-synuclein and autophagic flux, in Parkinson’s disease models^31^.

Taken together, observations of IRM across development, aging, and disease point to complex regulatory processes that underlie induction of IRM. This motivated us to systematically evaluate genetic regulators of the IRM state with a genome-wide CRISPR interference screen. We recovered a large proportion of canonical interferon signaling regulators and ISGs that regulate microglial access to the IRM state, confirming their importance in microglia. Additionally, we identified novel regulators of IRM, including a non-canonical role of the CCR4-NOT deadenylase subunit CNOT10. Since the direction of IRM regulation for therapeutic purposes may be context-specific, we have identified both inhibitors and activators of the IRM state. These regulators may be used to selectively perturb IRM in order to determine the function of this activation state in specific disease contexts, and may also be applied towards the development of therapeutic intervention for diseases in which the IRM state is relevant.

## Results Identification of interferon-responsive genes in iPS-Microglia

**Fig. 1:**
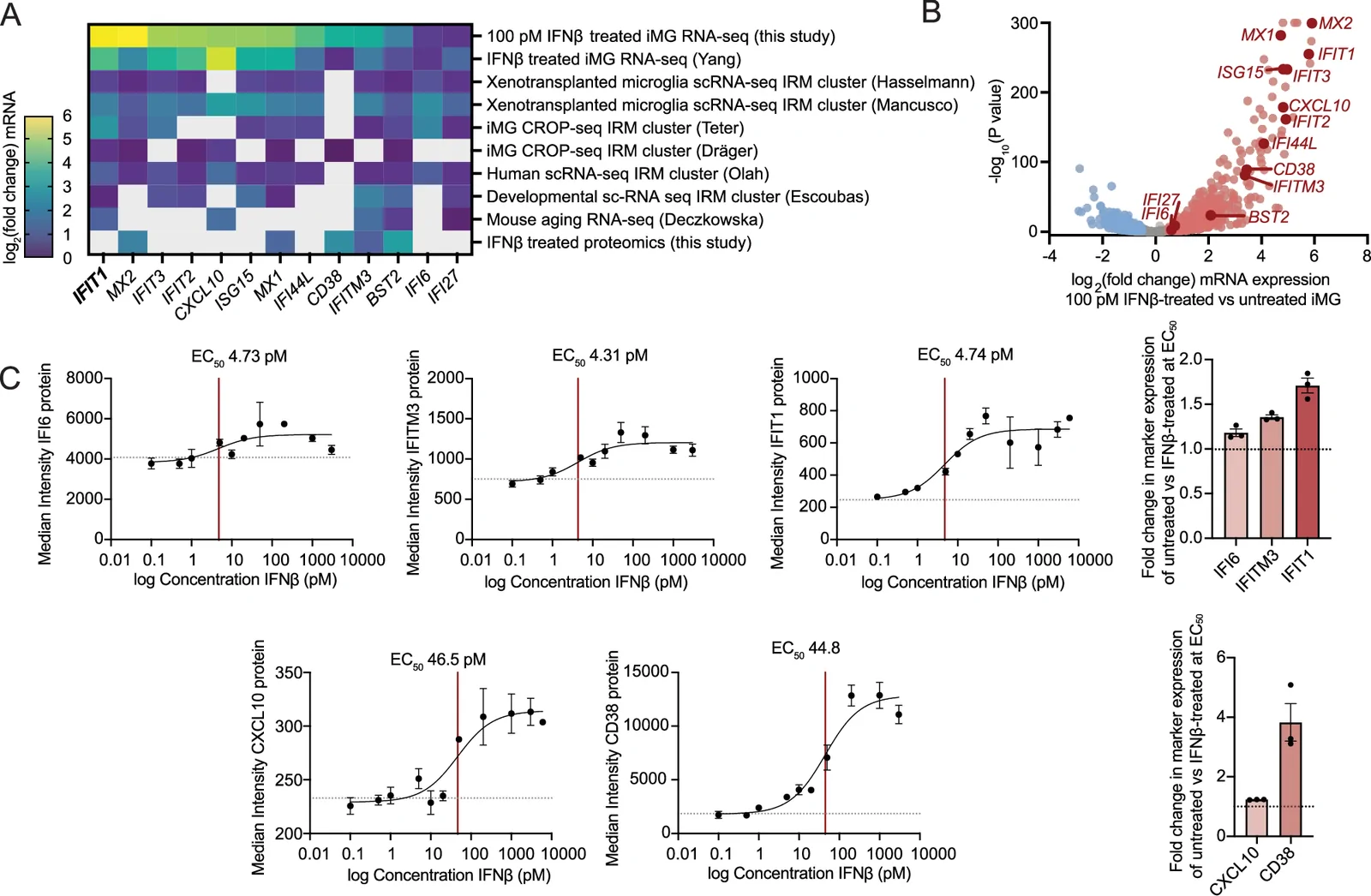
Identification of robust markers for human interferon-responsive microglia. **A** Comparison of mRNA expression across models of IRM. Log_2_(fold change) mRNA expression for 100 pM IFNβ vs untreated iPS-Microglia (bulk RNA-seq, this study), 100 U/mL IFNβ vs untreated iPS-Microglia (bulk RNA-seq, Yang et al.^32^), IRM cluster versus all other clusters (Single-cell seq, Hasselmann et al.^33^), IRM cluster vs all other clusters (Single-cell seq, Mancuso et al.^34^), IRM cluster versus all other clusters (CROP-seq, Teter et al.^23^), IRM cluster versus all other clusters (CROP-seq, Dräger et al.^36^), IRM cluster versus all other clusters (Single-cell seq, Olah et al.^35^), IRM cluster versus all other clusters (Single-cell, Escoubas et al.^13^), 3 month microglia versus 22 month old microglia (bulk RNA-seq, Deczkowska et al.^25^). Grey fill represents that the given gene is not significantly altered in the dataset. **B** Volcano plot showing differentially expressed genes in 100 pM IFNβ treated iPS-Microglia versus untreated. Conserved ISGs from **A** are denoted in dark red. **C** Dose response curves for IFI6, IFITM3, IFIT1, CXCL10, and CD38. Median fluorescence intensity (MFI) of each IRM marker was measured after 24 h of treatment with IFNβ. Dotted horizontal line represents MFI in untreated iPS-Microglia. Red vertical line represents the IFN concentration that yields 50% MFI response. Fold change of MFI for proteins responsive at 5 pM (top) or 50 pM (bottom) shown on right. Graphs represent mean±standard error. *n* = 3 independent wells, >10,000 cells per well. Data is shown as individual well datapoints on the right.

To enable a genome-wide CRISPR-based screen for modifiers of the type I interferon response in induced pluripotent stem cell-derived microglia (iPS-Microglia), we first identified IFNβ-responsive transcripts via bulk RNA-sequencing in iPS-Microglia. We treated iPS-Microglia with low (10 pM) or high (100 pM) concentrations of IFNβ (Fig. 1A,B, Supplementary Fig. 1, Supplementary Data 1). Although 100 pM treatment resulted in larger effect sizes than 10 pM, we found a significant correlation (R^2^ = 0.93) of the differentially expressed genes (DEGs) in each of these two treatment concentrations (Supplementary Fig. 1).

To identify the most robust markers of interferon-responsive microglia (IRM), we cross-referenced our interferon-responsive genes against several published datasets, prioritizing those that from human postmortem tissue or human iPSC-derived models when possible^13,23,25,32–36^ (Fig. 1A). Five IRM marker proteins from this data were selected as candidate markers for a genome-wide screen. For each candidate marker protein, response to IFNβ was measured by flow cytometry over a broad range of IFNβ concentrations (Fig. 1C). Interestingly, three candidates (IFI6, IFITM3, IFITM1) had an EC_50_ of ~5 pM IFNβ, whereas two candidates (CXCL10, CD38) had an EC_50_ of ~50 pM. This mirrors findings from single-cell RNA sequencing that we previously performed on iPS-Microglia, which revealed two distinct IRM clusters, only one of which showed elevated CXCL10 and CD38^36^. Because the markers that responded at ~5 pM IFNβ were more conserved across datasets, we wanted to maximize discovery from this class of interferon-response gene. Interferon-Induced Protein with Tetratricopeptide Repeats 1 (IFIT1) showed the best dynamic response range (Fig. 1C) and was chosen for our genome-wide screen. Importantly, IFIT1 has been identified as a specific marker of the type I IFN-response pathway^37^, lending further specificity to our screening results. We validated that IFIT1 and IFITM3 are selectively induced by IFNβ, and not IFNγ, in iPS-Microglia (Supplementary Fig. 2).

## Regulators of IFIT1 expression in iPSC-Microglia

**Fig. 2:**
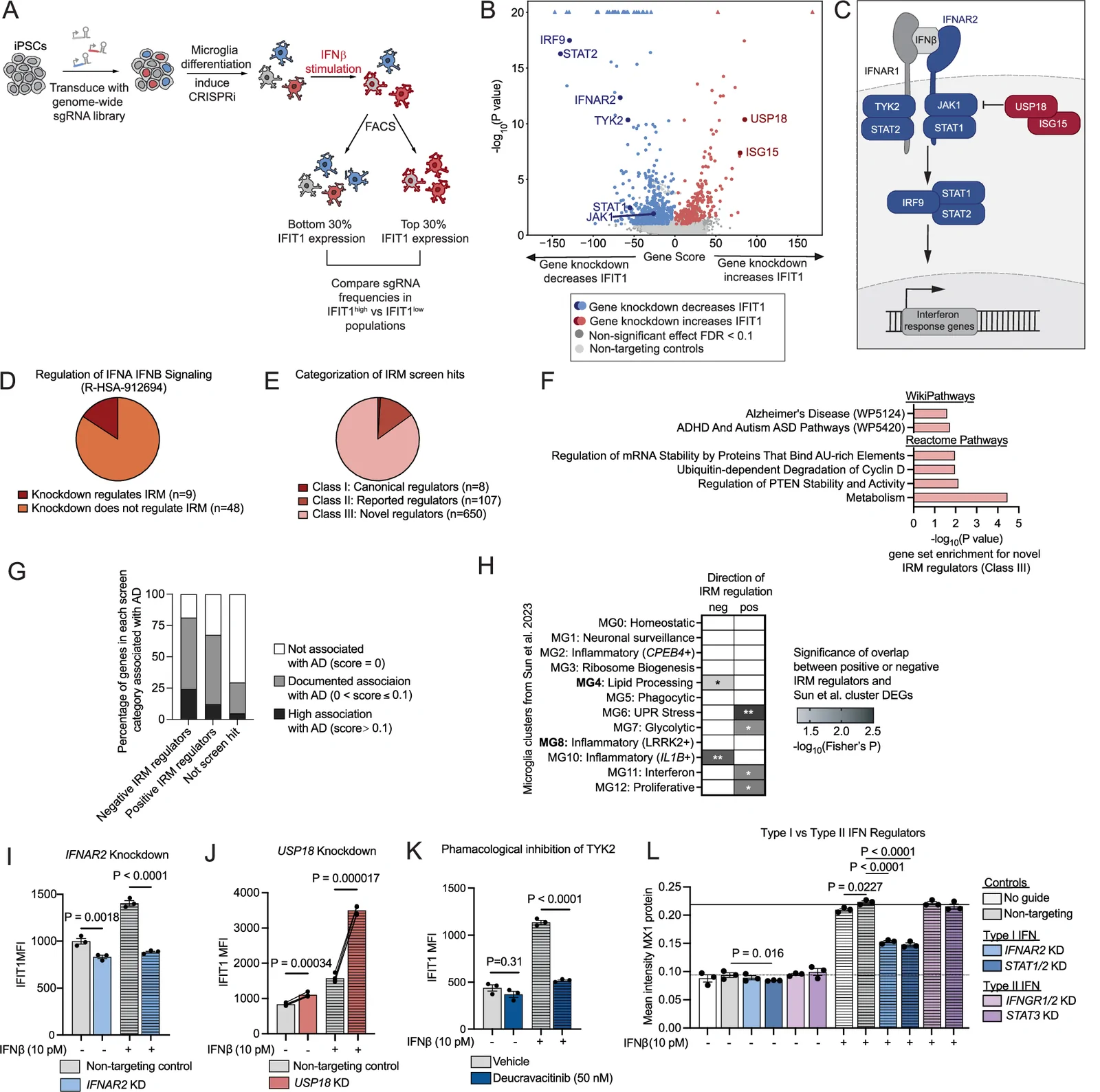
Interferon-responsive microglia are responsive to canonical interferon regulators. **A** Schematic of genome-wide screen for IFIT1 expression. iPSCs expressing inducible CRISPRi machinery were transduced sgRNAs targeting all genes in the genome at >1000x coverage. iPSCs were differentiated into microglia and CRISPRi knockdown was induced with trimethoprim (TMP). Microglia were stained with IFIT1 as a marker of the interferon-responsive microglia state. The 30% of cells with the lowest and highest expression of IFIT1 were separated by FACS. Genomic DNA was isolated from each population and the cassette encoding sgRNAs was amplified and sequenced using next-generation sequencing. Comparison of the frequencies of cells expressing each sgRNA to those expressing non-targeting control sgRNAs was used to determine hits. **B** Volcano plot of results from genome-wide screen. Gene score (normalized log_2_ ratio of sgRNA counts in IFIT1^high^ versus IFIT1^low^ populations) is plotted versus the negative log of the P value. Points represent the gene score for a single gene averaged across five individual sgRNAs targeting that gene. Positive hits (gene knockdown drives IFIT1) are plotted in red, negative hits (gene knockdown inhibits IFIT1) are plotted in blue. Non-hit genes are in grey. Quasi-genes generated from random sets of non-targeting control sgRNAs to model a null distribution are shown in lighter grey. Canonical interferon pathway genes are labelled. **C** Schematic of type I interferon signaling pathway. Positive hits are shown in red and negative hits are shown blue, non-hits are shown in grey. Blue and grey colored genes make up the Google Gemini canonical interferon response genes. **D** Overlap of screen hits with the Reactome pathway *Regulation of IFNA IFNB Signaling*. Pathway members that are screen hits are colored in red. Pathway members that were not significant in our screen are colored in orange. **E** Google Gemini classification of screen hits (see Methods) as canonical interferon regulators (darkest red, these genes are also shown in Fig. 2C in blue and grey), genes reported to be associated with interferon regulation (red) or novel interferon regulators (light red). **F** Gene set enrichment analysis of genes in Class III (novel regulators) is shown for selected WikiPathways and Reactome pathways. See also Supplementary Data 6. P value of enrichment calculated by IDEA. **G** Open Targets^40^ AD-association gene scores for genes we detected as negative, positive, or not regulators of IRM. Scores are stratified by No association (score = 0, white), documented association (0 < score ≤ 0.1, light grey), or high association (score > 0.1, dark grey). Y axis represents the percentage of the total genes that are negative, positive, or not regulators of IRM in each Open Targets score category. **H** Heatmap of gene signature overlap analysis of positive and negative IRM regulators with human microglia clusters from Sun et al. 2023^41^. Bolded states are increased in AD patients. Color scale denotes significance of overlap by Fisher’s exact test P value. Non-significant overlap is white. *P < 0.05, **P < 0.01. **I** Median fluorescence intensity (MFI) of IFIT1 protein levels in *IFNAR2* knockdown (blue) versus non-targeting control cells (grey). 10 pM IFNβ was added for 24 h before flow cytometry analysis. (*n* = 3 independent wells > 10,000 cells per replicate. Bars represent mean±standard error, T test) **J** Median fluorescence intensity (MFI) ±standard error of IFIT1 protein levels in *USP18* knockdown (red) versus non-targeting control (grey) cells plated as in-well controls. 10 pM IFNβ was added for 24 h before flow cytometry analysis. *N* = 3 independent wells, > 10,000 cells per replicate. Bars represent mean±standard error, paired T test. **K** Median fluorescence intensity (MFI) of IFIT1 protein levels after Deucravacitinib (blue) versus vehicle (grey). After 30 min pre-treatment with Deucravacitinib or vehicle, 10 pM IFNβ was added for 24 h before flow cytometry analysis. *n* = 3 independent wells, > 10,000 cells per replicate. Bars represent mean±standard error, T test. **L** Mean fluorescence intensity of MX1 protein levels normalized to DAPI nuclear counts after knockdown versus non-targeting control. 10 pM IFNβ was added for 24 h before fixation. *n* = 3 independent wells averaging from 4 images per well, Bars represent mean±standard error, ANOVA with Tukey’s multiple comparison test.

To determine regulators of IRM, we performed a genome-wide CRISPRi screen based on expression of IFIT1. Microglia were pre-stimulated with 10 pM IFNβ for 24 h to induce the IRM state and sorted based on expression of IFIT1 (top or bottom 30% of cells based on IFIT1 expression) (Fig. 2A). We identified 772 genes that regulated IFIT1 expression (Fig. 2B, Supplementary Data 2). Of the 772 hit genes, we found 273 negative regulators (knockdown increases levels of IFIT1) and 499 positive regulators (knockdown decreased levels of IFIT1). Top hits for which knockdown negatively regulated interferon response include canonical interferon signal transduction pathway effectors (*TYK2, IFNAR2, JAK1, STAT1, STAT2, IRF9*) (Fig. 2B,C, Supplementary Data 2). In addition, we identified that knockdown of canonical negative regulators of interferon signal transduction permitted higher interferon response (*ISG15, USP18)*. Interestingly, knockdown of homeostatic signaling receptors (*TGFBR1, TGFBR2)* also promoted IFIT1 levels after IFNβ stimulation, suggesting that these proteins normally act to attenuate IFNβ signaling.

We performed gene set enrichment analysis of the full set of IRM regulators nominated by our screen using the Reactome Pathways 2024 library. We found significant enrichment of pathways related to interferon-response including *Cytokine Signaling in Immune System, Regulation of IFNG Signaling, Regulation of IFNA IFNB Signaling, Interferon Alpha Beta Signaling, and Interferon Alpha Beta Signaling* in our list of IRM regulators (Supplementary Data 3). It is important to note that for each of these families, our screen only identified a subset of the predicted regulators (Supplementary Data 3, Fig. 2D). Given that our screen perturbed one gene at a time, it is biased towards the discovery of regulators that are effective when targeted individually. Regulators with high levels of redundancy could not be discovered with this screen design. Because of this design, this screen can also be used to prioritize nodes with therapeutic potential.

Our gene set enrichment analysis also revealed enrichment of pathways not directly tied to interferon signaling or viral response. We found significant enrichment of *Deadenylation of mRNA, Regulation of PTEN Stability and Activity*, and several pathways related to TP53 including *Transcriptional Regulation by TP53, TP53 Regulates Transcription of Cell Cycle Genes*, and *p53-Dependent G1 DNA Damage Response* (Supplementary Data 3). TP53 was discovered as a positive regulator of IRM (knockdown decreases IFIT1) in our screen. One previous study nominated IFIT1 as a direct target of TP53^38^, although this has not been replicated across multiple datasets^39^. However, other IRM genes have also been nominated as TP53 targets including IFIT3, MX2, OAS1, IFNAR2, TBK1, IRF1, IRF5, IRF9^39^. This further supports our findings that TP53 may regulate IRM.

To determine if there are specific transcription factors that may collectively drive the gene sets discovered in our screen, we performed gene set enrichment from the TRRUST database. We found that negative hits were enriched for predicted STAT1 and STAT2 regulons (TRRUST v2; FDR STAT1: 0.0001, STAT2: 0.0357). Our positive hits were enriched for predicted SP1 and IRF2 regulons (TRRUST v2; FDR SP1: 0.0084, IRF2: 0.0212).

As interferon regulation in peripheral systems has been under focused research for decades, we mined existing research studies using the Google Gemini large language model to determine the extent to which our hits have previously been linked to interferon signaling. Google Gemini was used to classify our screen hits into “canonical interferon regulators”, which include those directly downstream of IFNβ (Fig. 2C, labelled in blue and grey), genes with a “reported association with interferon”, which include genes found in IFN- and viral-response gene ontology terms, as well as those for which one or more publications mention the gene in the context of IFN, and “novel interferon regulators”, for which Google Gemini did not find any known associations with IFN response (Fig. 2E, Supplementary Data 4,5). The majority of hit genes from our screen were categorized into the third category of novel interferon regulators. To better understand the novel interferon regulators revealed by our screen, we performed gene set enrichment analysis of only the Class III novel gene set. We found enrichment of several Reactome pathways related to mRNA stability, cell cycle, and metabolism (Fig. 2F, Supplementary Data 6). We also found significant enrichment of two disease pathways from WikiPathways_Human_2024: *Alzheimer’s Disease (WP5124)* and *ADHD and Autism ASD Pathways (WP5420)* (Fig. 2F, Supplementary Data 6).

To interpret the relationship between our IRM regulators and Alzheimer’s disease (AD) more robustly, we mined the Open Targets^40^ database to determine the extent to which our screen hits are associated with AD. Open Targets assigns a disease score for every gene based on accumulating evidence that a specific gene is associated with a specific disease. We found significantly higher AD gene scores for our IRM regulators compared to non-hit genes (Mann-Whitney U test P value 7.5×10^-154^). We further stratified our Open Targets disease scores into high AD-association (score > 0.1), documented AD-association (0 < score ≤ 0.1), or no AD-association (score = 0). Our screen hits were enriched for both highly associated and moderately associated AD-genes. We found this to be true for both positive and negative IRM regulators, which is not surprising given that the Open Targets gene scores are agnostic of the direction of the association (risk vs protection from disease) (Fig. 2G).

To understand the direction of the relationship between IRM regulators and Alzheimer’s disease, we analyzed differences in expression of our hit genes in AD-patient derived microglia. Since brain-derived microglia exist as a heterogeneous population, an enrichment or depletion of specific transcriptomic clusters or states is often used, rather than global up-regulation or down-regulation. We first determined the overlap of our IRM regulators with microglia clusters defined by Sun et al.^41^ from human patient-derived microglia (Fig. 2H). As expected, we found that our positive IRM regulators (knockdown decreases IFIT1) overlap with MG11, an interferon signaling gene module. We also found overlap of positive IRM regulators with MG6, MG7, and MG12 corresponding to unfolded protein response, glycolytic, and proliferative gene modules. Our negative IRM regulators (knockdown drives IFIT1) showed significant overlap with the MG4 cluster, which is involved in lipid processing as well as MG10, a neuroinflammatory module. Sun et al. showed that the MG4 cluster specifically is enriched in microglia from AD patients. This suggests that our negative regulators of IRM are enriched in AD patient brains. Further research will be required to understand the exact impact that interferon-responsive microglia may have on these disease pathways and cellular responses.

## Canonical regulation of interferon is relevant in iPS-Microglia models

To validate the results from our screen, we generated independent CRISPRi knockdown lines targeting canonical interferon pathway genes (knockdown validation in Supplementary Fig. 3). Corroborating the results of our screen, we found that knockdown (KD) of the type I interferon receptor, *IFNAR2*, in iPS-Microglia abolishes the response to interferon (Fig. 2I). Interestingly, we found that microglia lacking *IFNAR2* showed decreased IFIT1 levels even at baseline, suggesting that this model of iPS-Microglia has some level of tonic IFNβ production. This corroborates previous findings highlighting the existence of IRM at baseline by single-cell RNA sequencing analyses^23,36,42^.

Ubiquitin Specific Peptidase 18 (USP18) competitively inhibits JAK1 binding to IFNAR2 and blocks the necessary dimerization of IFNAR1 and IFNAR2. Knockdown of this negative regulator of interferon signaling increased IFIT1 levels both at baseline and after interferon stimulation (Fig. 2J). Knockdown of *USP18* increased iPS-Microglia response to IFNβ by over two-fold, suggesting that USP18 may be a useful node to drive type I IFN responses therapeutically. We previously identified that knockdown of Interferon-Stimulated Gene 15 (*ISG15*), also increased IFIT1 expression, although the effect size was smaller in microglia with *ISG15* knockdown compared to microglia with *USP18* knockdown^42^.

Tyrosine Kinase 2 (TYK2) associates with IFNAR1/2 to phosphorylate the Signal Transducer and Activator of Transcription 1 and 2 (STAT1 and STAT2) transcription factors that are necessary for transcription of interferon-responsive genes, including IFIT1. As expected, in our iPS-Microglia system, IFNβ-induced expression of IFIT1 is blocked by the TYK2 inhibitor, Deucravacitinib, though no difference in IFIT1 levels was detected in the absence of IFNβ stimulation (Fig. 2K).

Completing our analysis of canonical interferon pathway regulation, we have also recently demonstrated that knockdown of any of the ISG3 complex members (*STAT1, STAT2, and IRF9)* in iPS-Microglia reduce interferon response at the transcriptomic level^42^.

Both Type I and Type II interferon have been implicated in the progression of Alzheimer’s disease, with plaque-associated microglia showing high levels of IFIT1 and MX1^11,16,43^. Using CRISPRi knockdown of canonical Type I and Type II interferon signal transduction genes, we showed that MX1 is responsive to IFNβ stimulation and that this could be specifically blocked with knockdown of Type I IFN pathway genes (*IFNAR2, STAT1/2*) but not Type II IFN (*IFNGR1/2, STAT3*) (Fig. 2L).

## CCR4-NOT Transcription Complex Subunit 10 (CNOT10) knockdown inhibits IRM

**Fig. 3:**
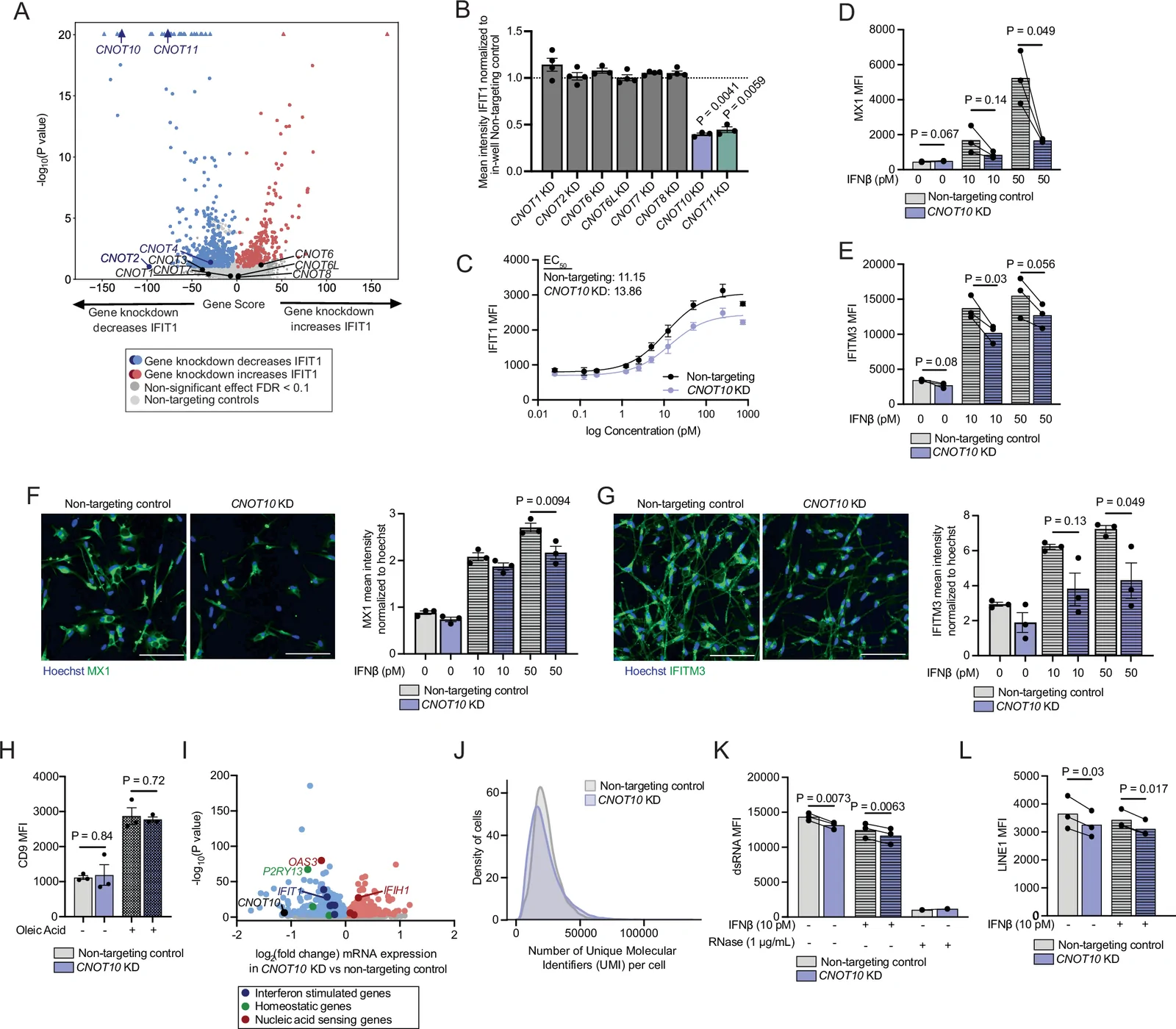
Knockdown of *CNOT10* inhibits IRM. **A** Volcano plot of results from genome-wide screen. Gene score (normalized log_2_ ratio of sgRNA counts in IFIT1^high^ versus IFIT1^low^ populations) is plotted versus the negative log of the *P* value. Points represent the gene score for a single gene averaged across five individual sgRNAs targeting that gene. Positive hits (gene knockdown drives IFIT1) are plotted in red, negative hits (gene knockdown inhibits IFIT1) are plotted in blue. Non-hit genes are in grey. Quasi-genes generated from random sets of non-targeting control sgRNAs to model a null distribution are shown in lighter grey. All members of the CCR4-NOT complex are labelled (significant genes in blue, non-significant genes in black). **B** Median fluorescence intensity (MFI) of IFIT1 protein levels in CCR4-NOT family normalized to non-targeting in-well controls (*n* = 3-4 independent wells, > 10,000 cells per replicate. Bars represent mean±standard error, T test). **C** Dose response curves for interferon response in *CNOT10* knockdown (periwinkle) vs non-targeting control (grey). Median fluorescence intensity (MFI) of IFIT1 was measured after 24 hours of treatment with IFNβ. Data represent mean±standard error. *n* = 3 independent wells > 10,000 cells per well. EC_50_ calculated as nonlinear fit [agonist] vs response in Prism. **D**, **E** Median fluorescence intensity (MFI) of **D** MX1 and **E** IFITM3 protein levels in *CNOT10* knockdown (periwinkle) verses in-well, non-targeting control (grey) sgRNA. IFNβ was added at the indicated concentration for 24 h prior flow cytometry analysis. *n* = 3 independent wells, > 10,000 cells per replicate. Bars represent mean±standard error, ANOVA, Tukey’s multiple comparison test. **F**, **G** Median intensity of **F** MX1 and **G** IFITM3 by immunofluorescent imaging. Representative images are shown of Hoechst nuclear stain (blue) and MX1 or IFITM3 (green). Scale bar = 10 µm. Quantification of MX1 or IFITM3 intensity per cell is normalized to nuclear intensity. *N* = 3 wells with 4 images averaged per well. P value from One-way ANOVA. (H) Median fluorescence intensity (MFI) CD9 surface protein levels in *CNOT10* knockdown (periwinkle) versus non-targeting control (grey). 250 nM Oleic acid was added 24 h prior to flow cytometry analysis. *N* = 3 independent wells, > 10,000 cells per replicate. Bars represent mean±standard error, ANOVA, Tukey’s multiple comparison test. **I** Volcano plot of differentially expressed genes in *CNOT10* KD versus non-targeting control cells. Genes that increase or decrease in expression in *CNOT10* KD are colored red or blue (FDR ≤ 0.05). Genes related to interferon, microglia homeostatic markers, or nucleic acid sensing are highlighted. **J** Number of unique molecular identifiers (UMI) per cell from PIP-seq experiment of microglia with *CNOT10* KD or non-targeting control sgRNA *n* = 2 separately prepared samples. Mann-Whitney U test *P* = 9.6*10^-30^. **K** Median fluorescence intensity (MFI) of double-stranded RNA (dsRNA) measured by flow cytometry in iPS-Microglia with *CNOT10* knockdown or in-well, non-targeting control sgRNA. For the indicated bars, 10 pM IFNβ was added for 24 h before fixation or 1 µg/mL RNase A for 30 min after fixation. Bars represent mean±standard error, Paired t-test. For non-targeting control and *CNOT10* KD comparison, *n* = 3 independent wells, > 10,000 cells per well. For RNase control, *n* = 1 well, > 10,000 cells per well. **L** Median fluorescence intensity (MFI) of LINE1 DNA measured by flow cytometry in iPS-Microglia with *CNOT10* knockdown (periwinkle) or in-well, non-targeting control (grey) sgRNA. 10 pM IFNβ was added for 24 h to the indicated bars. *n* = 3 independent wells, > 10,000 cells per well. Bars represent mean±standard error, Paired t-test.

Two of our most significant screen hits for which knockdown decreases IFIT1 are CCR4-NOT Transcription Complex Subunit 10 and 11 (*CNOT10, CNOT11*). CNOT10 and CNOT11 are both subunits of the CCR4-NOT transcription complex that is responsible for deadenylating mRNA^44–46^ (Fig. 3A). While we found that the Reactome pathway *Deadenylation of mRNA* was significantly enriched (FDR = 0.00187) in our IFIT1 screen (Supplementary Data 3), it is important to note that none of the enzymatic deadenylase subunits (*CNOT6, CNOT6L, CNOT7, CNOT8)* or the scaffold of the complex (*CNOT1)* were discovered as IRM regulators by our screen. We independently validated this result by flow cytometry using individual sgRNA knockdown lines. Only *CNOT10* and *CNOT11* KD decreased IFIT1 expression (Fig. 3B, C, Supplementary Fig. 4), suggesting that the canonical deadenylation functions of this complex do not explain the difference in interferon response seen in *CNOT10* and *CNOT11* KD microglia. We confirmed that *CNOT10* knockdown inhibited IRM using multiple markers of the state and a wide range of concentrations (Fig. 3 C-G, Supplementary Fig. 4). Our data suggests that *CNOT10* KD microglia have a maximal response to interferon that is lower than their WT counterparts and a right-shifted EC_50_ of IFIT1 response. We found that knockdown of *CNOT11* is less consistent across multiple markers of the IRM state (Supplementary Fig. 4) and therefore focused our analysis on *CNOT10*.

Because CNOT10 is part of a complex that de-adenylates mRNA, knockdown of CNOT10 could be globally altering mRNA stability and impacting microglial activation towards numerous states. If mRNA was destabilized broadly, we would anticipate that *CNOT10* KD microglia would be unable to activate into any activation states. We therefore tested the specificity of our IRM phenotype by staining for markers of other microglial activation states including the disease-associated (CD9, TREM2), chemokine-secreting (CCL2, CCL13), homeostatic (P2RY12), and antigen-presenting (HLA-DMB, CD74) states. We found that none of these state markers are inhibited in the *CNOT10* or *CNOT11* knockdown microglia compared to cells with non-targeting control sgRNA (Supplementary Fig. 5). When driven towards the disease-associated state with oleic acid, both non-targeting control sgRNA and *CNOT10* KD iPS-Microglia activated CD9 expression to the same extent (Fig. 3H).

Due to scale, our original genome-wide screen was only performed in microglia derived from one iPSC line and used CRISPRi knockdown beginning early in microglia differentiation to produce the most robust knockdown effects^36^ and maximize discovery of IRM regulators. To test if our *CNOT10* KD phenotypes robustly reproduce in microglia derived from an independent iPSC line, using an orthogonal knockdown method (RNAi), and knocking down our target after microglia maturation, we performed additional experiments. With these orthogonal methods, we confirmed that *CNOT10* KD microglia have lower levels of the ISGs IFIT1, MX1, and IFITM3 than cells transduced with control siRNA (Supplementary Fig. 6A-D). We also confirmed the specificity of inhibition of the IRM state as markers of other activation states are not decreased after *CNOT10* RNAi knockdown (Supplementary Fig. 6E-H).

To examine the impact of *CNOT10* knockdown on mRNA, microglial activation and interferon-response more closely, we performed single-cell RNA-sequencing on *CNOT10* KD or non-targeting control microglia after 24 h of IFNβ stimulation (Fig. 3I, Supplementary Data 7). Because CCR4-NOT is involved in mRNA stability, we compared the unique molecular identifiers (UMI) per cell from the *CNOT10* KD microglia compared to non-targeting control cells. Interestingly, we did find a small but significant decrease in UMI per cell from the *CNOT10* KD microglia compared to non-targeting control cells (Fig. 3J). This may indicate that *CNOT10* KD microglia do have lower levels of polyadenylated mRNA captured by Particle-templated Instant Partitions sequencing (PIP-seq). However, this effect size was minimal and not indicative of broad scale mRNA disruption. It is not completely unexpected that mRNA deadenylation continues to function in the absence of CNOT10, given that other organisms, such as yeast, have no ortholog of CNOT10 but maintain CCR4-NOT function^44,47^.

Differential expression of mRNA from CNOT10 KD microglia and non-targeting controls showed approximately equal numbers of differentially expressed genes in the positive and negative direction (403 increased expression, 506 decreased expression in *CNOT10* KD), supporting our hypothesis that IRM inhibition is not merely a byproduct of generally decreased mRNA. Corroborating our protein-level findings, mRNA levels of multiple interferon-stimulated genes were decreased in *CNOT10* KD microglia, specifically *IFIT1, IFIT3, IFI6*, *IFI30, IFIT5*, and *IFITM1* (Fig. 3I, Supplementary Data 7). Expression of *S100B* was also lost in *CNOT10* KD microglia, a secreted factor that has recently been shown to be critical for binding of IFNβ to IFNAR1/2^48^. Of interest, there were expression changes in several genes involved in nucleic acid sensing, an important driver of IRM. Transcript levels of nucleic acid sensors Cyclic GMP-AMP Synthase (*CGAS)* and Interferon-Induced with Helicase C Domain 1 (*IFIH1)*, encoding MDA5, were surprisingly increased in *CNOT10* KD microglia. However, the upstream dsRNA-binding protein 2’, 5’ oligoadenylate (*OAS3)* was decreased in expression in *CNOT10* KD microglia. Although it would be unexpected given that *CNOT10* KD microglia have lower interferon-response, we assessed if there is evidence of increased dsRNA in *CNOT10* KD microglia and did not find evidence to support this (Fig. 3K). Additionally, we found no evidence of increased LINE1 DNA, another inducer of interferon-responses through nucleic acid sensing pathways (Fig. 3L).

Further supporting our protein level analysis of *CNOT10* KD, our RNA-sequencing data showed no difference in mRNA expression of the activation state markers *CD9, TREM2*, or *CCL13*. Interestingly, our RNA-sequencing data revealed lower levels of microglia homeostatic markers, *CX3CR1*, *P2RY12* and *P2RY13* (Fig. 3I) despite *CNOT10* KD microglia not seeming robustly activated. We found no change in expression of P2RY12 at the protein level (Supplementary Fig. 5). To further ensure that the decreased homeostatic markers at the mRNA level was not indicative of a loss of microglia identity, we stained *CNOT10* KD microglia for CD45, CD11b, PU.1 and IBA1 and did not find decreased levels of these markers compared to the non-targeting control cells (Supplementary Fig. 7).

Importantly, expression of *CNOT10* is decreased at the mRNA level in our *CNOT10* KD lines, while expression of all other CCR4-NOT complex subunits remain unchanged (Supplementary Data 7). Since the CCR4-NOT complex can function without CNOT10^49,50^, it is likely that our knockdown lines remain competent in deadenylation. However, *CNOT10* KD microglia showed changes to gene expression that could impact global translation. On the one hand, genes encoding subunits of both the small and large ribosomal proteins were enriched among DEGs with decreased expression in *CNOT10* KD microglia. On the other hand, many translation initiation factors were increased in expression in the *CNOT10* knockdown microglia. TATA-box binding protein associated factor, RNA polymerase I subunit B (*TAF1B*), which contributes to transcription of ribosomal RNA, was also increased in expression in *CNOT10* KD cells. Together, these results suggest there may be changes in translation, though it remains unclear how this would impact specific microglial activation states (IRM) and not others (disease-associated, homeostatic, chemokine-secreting, antigen-presenting) as our protein-level analysis showed.

## Discussion

In this study, we present a genome-wide CRISPR interference screen for expression of the Type I interferon marker IFIT1. An initial investigation of the concentration-dependent responses of interferon-stimulated genes (ISGs) at the transcriptomic level revealed high correlation between ISG induction at 10 pM vs 100 pM IFNβ, suggesting that there are not two distinct response profiles at the two concentrations. However, at the protein level, a subset of ISGs including CXCL10 and CD38 showed a median response to IFNβ at 50 pM, whereas the majority of ISGs peaked at a lower IFNβ concentration ( ~ 5 pM). In future work, it may prove useful to understand the functional differences between low IFN-responsive profiles and high IFN-responsive profiles to determine if there are two classes of interferon-responsive microglia or if higher concentrations merely exacerbate the same functions.

The IFIT1-based screen presented here relies on an acute interferon treatment, which may be more reflective of a viral infection or the earlier stages of neurodegeneration. In tauopathies, it has been shown that type I interferon responses are particularly enriched early in disease^16^. Our genome-wide IRM screen does not test microglial responses to chronic neuroinflammatory conditions like long COVID or multiple sclerosis. Future research is required to test our nominated activators/inhibitors in chronic interferon-high environments. This could be modeled in vitro with repeated interferon stimulation over several weeks, or in vivo by pairing models of neuroinflammation or neurodegeneration with microglia-specific gene knockdown.

The set of regulators identified in our genome-wide screen supports the hypothesis that IRM are regulated via canonical interferon signal transduction. This finding suggests that tools developed to combat peripheral interferon- and viral-responses can be translated for use in the central nervous system. For example, we highlight that knockdown of *STAT1, STAT2, IFNAR2, TYK2*, and *USP18* modulate IFIT1 expression in microglia in the expected direction. Unexpectedly, our original screen did not highlight IFNAR1, a co-receptor for IFNβ, as a modifier of IFIT1. Upon closer analysis, we determined that our *IFNAR1*-targeting sgRNAs did not perturb *IFNAR1* at the mRNA level (Supplementary Figure 3C), explaining this result. Lastly, we show that treatment with Deucravacitinib, the FDA-approved inhibitor of TYK2, blocks interferon responses in iPS-Microglia. Moving forward, our full catalog of predicted IRM regulators can be used to nominate other existing, FDA-approved therapeutics that could perturb microglial interferon responses.

There is a high degree of redundancy in the interferon-response pathway. For example, there are 13 proteins in the IFNα family, multiple members of the Protein Inhibitors of Activated STAT (PIAS) family, as well as multiple ubiquitin-encoding genes, and E3 ligases that negatively regulate interferon signaling. While our screen hits did show significant (FDR = 7.11E-5) gene set enrichment of the Reactome pathway *Regulation of IFNA IFNB Signaling*, only 9 of the 57 genes in this pathway were significant regulators in our screen. These 9 screen hits (*IFNAR2, TYK2, JAK1, STAT1, STAT2, USP18, PTPN1, PTPN6*, and *SOCS3)* must therefore have non-redundant functions in microglia since we were able to regulate IRM through knockdown of each individual node. In contrast, the remaining 48 genes in this Reactome pathway may be buffered by significant redundancies as described above. Importantly, this distinction highlights a key feature of our screening approach in that it prioritizes discovery of non-redundant regulatory nodes that are inherently more tractable for therapeutic development.

Intriguingly, for one of these canonical regulators, Protein Tyrosine Phosphatase 1B (PTPN1), we report regulation of IRM opposite to the expected direction. PTPN1 is known to dephosphorylate JAK1 and TYK2, acting as a negative regulator of interferon-signaling. However, it has also been reported that *PTPN1* knockout macrophages secrete lower levels of type I IFN^51^, suggesting that PTPN1 positively regulates the second wave of interferon signaling. This function is independent from its phosphatase activity and requires localization to the ER. Our screen uncovered PTPN1 to be a positive regulator of IFIT1 expression in microglia (knockdown of *PTPN1* lowers IFIT1), providing evidence to support this non-canonical function. PTPN1 has been an attractive therapeutic target in type II diabetes, obesity, and cancer for its roles in insulin sensitivity, leptin resistance, and inflammation^52–54^. Our work suggests that PTPN1 inhibitors could also be adapted for use in the brain to reduce interferon-responsive microglia. Cen et al.^55^ have recently tested this hypothesis by using the small molecule DPM-1003 to inhibit PTPN1 in a murine model of Alzheimer’s disease. They found beneficial effects on plaque load and memory, and corroborated our finding that blocking PTPN1 in microglia increases the levels of ISGs^55^.

In addition to the identification of canonical interferon-signaling genes, our screen has revealed a large number of novel regulators of IRM as well. Interestingly, we found that many genes from loci associated with Alzheimer’s disease (AD)-risk altered levels of IFIT1. From 1077 genes in AD risk loci, 128 were hits in our genome-wide screen. For 101 genes out of the 128, targeting with CRISPRi drives interferon-response. However, it remains unclear what this means for Alzheimer’s pathology, since many of the AD-risk loci have not been mapped to a particular causal gene, and even for those with causal genes proposed, the functional impact of the SNP is not well understood.

Of particular interest, we found that knockdown of APOE and INPP5D increased levels of IFIT1. Previous studies have shown that APOE ε4 variant carriers have higher levels of IFNβ in the serum and plasma^27^. Likewise, APOE ε4 itself has been identified as a ligand for Leukocyte Immunoglobulin-Like Receptor B3 (LILRB3) which triggers a downstream interferon response whereas APOE ε2 does not produce this response^56^. Similarly, studies have found INPP5D to negatively regulate interferon through binding of IRF3 in models of viral infection. Whether this mechanism underlies the impact of INPP5D on neurodegenerative disease remains unclear.

We also discovered CD33 and PTPN6 negatively regulate IRM. CD33 is a sialic acid-binding immunoglobulin-like lectin (siglec) that signals through its immunoreceptor tyrosine-based inhibitory motif (ITIM) to restrict activation. CD33 can regulated through interaction with two key phosphatases PTPN6 and PTPN11. Of these two phosphatases, only PTPN6 has been shown to be associated with AD-risk^57^. We find that only CD33 and PTPN6 negatively regulate IRM, not PTPN11, mirroring the specificity of the relationship with AD-risk. This evidence supports the hypothesis that mutations in CD33 and PTPN6 may impact AD-risk through inhibition of the IRM state. PTPN6 was also recently shown to impact AD pathology in murine models by regulating the DAM state, though the bulk RNA-sequencing did detect increased levels of ISGs after *PTPN6* knockout^58^. Future research will be required to parse the impact these two disease-relevant activation states on brain health.

Two of our top novel regulators of IRM are CCR4-NOT Transcription Complex subunits 10 and 11 (*CNOT10, CNOT11*). CNOT10 and CNOT11 were identified in 2013 as novel subunits of the eukaryotic CCR4-NOT complex. These subunits evolved more recently than the rest of the CCR4-NOT complex and have no orthologues in yeast^45^. While CNOT10 and CNOT11 do contribute to canonical functions of CCR4-NOT deadenylation, particularly helping with ribosomal binding^59^, CNOT10 and CNOT11 have also been shown to interact with Gametogenetin Binding Protein 2 (GGNBP2)^45,60^. This interaction is proposed to influence nucleic acid recognition pathways by re-localizing native dsRNA that has not been edited by Adenosine Deaminase RNA-Specific (ADAR) away from the cytoplasm, resulting in decreased interferon response^60^. Heraud-Farlow and colleagues show that knockdown of *CNOT10* or *CNOT11* in myeloid cells limits interferon response, which directly matches the effect we observed. However, Heraud-Farlow et al. only find this interaction to occur in ADAR editing mutants, while our iPS-Microglia are ADAR competent. Additionally, the changes in dsRNA and LINE1 DNA levels in our *CNOT10* knockdown microglia are small in effect size. Still, this may suggest that tonic interferon signaling in this iPS-Microglia model is in part driven by nucleic acid recognition. In opposition, Gordon et al. found *CNOT10* KD amplifies interferon responses, leading to more potent antiviral activity. This study was performed with HeLa cells and primary CD4 + T cells, which may explain the difference in findings^61^. It is possible that the effects we and Heraud-Farlow have found are specific to the myeloid lineage, though further research is required to address this hypothesis.

Due to the scale of our genome-wide screen, we were limited to use microglia derived from one patient background, and one marker of the IRM state. In our studies of CNOT10 and CNOT11, we tested the IRM state with additional markers and in an additional patient background. These careful studies should be applied to other novel IFIT1 regulators before their impact on the IRM state can be concluded. Another limitation of the genome-wide screen is that genes were knocked down during microglia differentiation. It is possible that some of our discovered hits impact IFIT1 expression by derailing microglia differentiation, though we did not find direct evidence of this. We have previously shown^36^ that inducing knockdown after cellular differentiation limits the efficacy of CRISPR inhibition, likely due to changes to the chromatin state. However, due to this design, we cannot exclude the possibility that some IFIT1 regulators identified here merely alter microglia differentiation. For CNOT10 and CNOT11, we confirmed that expression of microglia identity genes is not lost.

In summary, our results define a catalog of canonical and noncanonical regulators of the interferon-responsive microglial activation state and point to nodes within the interferon-response pathway that could be harnessed to tune microglia function across diverse developmental and disease contexts.

## Methods

### iPSC maintenance

Human iPSCs (WTC11 or KOLF2.1 J background with doxycycline-inducible transcription factors: CEPBA, CEBPB, IRF5, IRF8, MAFB, PU.1) were cultured in StemFlex (Gibco, A33493-01) on Matrigel (Corning, 356231) coated plates. Media was changed every day and cells were passaged once per week using ReLeSR (100-0483). Human iPSC studies at the University of California, San Francisco were approved by the Human Gamete, Embryo and Stem Cell Research Committee.

### Differentiation of iPS-Microglia

iPS-Microglia were differentiated as previously described^36^ with a modified media composition optimized for increased expression of P2RY12, TREM2, and CD33^42^. iPSCs containing six doxycycline-inducible transcription factors were dissociated to a single-cell suspension with accutase (Thermo Fisher Scientific, A11105-01) for 7 minutes at 37 °C. and passaged onto dual coated Matrigel (Corning, 356231) and PDL plates (Corning, 356470). After culturing for 48 h in Essential 8 (Gibco, A1517001) stem cell maintenance media supplemented with 2 µg/mL doxycycline (Clontech, 631311) and 10 µM Y-27632 (Tocris 1254), cells were changed to microglia differentiation media: BrainPhys (StemCell Technologies, 05790), 0.5x N2 Supplement (Gibco, 17502048), 0.5x B27 (Gibco, 17504044), 10 ng/mL NT-3 (Peprotech, 450-03), 10 ng/mL BDNF (Peprotech, 450-02), 1 µg/mL mouse laminin (Thermo Fisher Scientific, 23017-015) supplemented with 2 µg/mL doxycycline, 100 ng/mL IL-34 (Peprotech 200-34), and 10 ng/mL GM-CSF (Peprotech 300-03). On day 4, day 8, and day 12, media was replaced with microglia differentiation media supplemented with 2 µg/mL doxycycline, 100 ng/mL IL-34, 10 ng/mL GM-CSF, 50 ng/mL M-CSF (Peprotech 300-25), and 50 ng/mL TGFB (Peprotech100-21C). For experiments with CRISPRi, cells were supplemented with 50 nM TMP (MP Biomedical, 195527) every other day to maintain strong knockdown.

### Flow cytometry

After 13 days of differentiation, iPS-Microglia were lifted with TrypLE (Gibco, 12604021) for 10 min at 37 °C. Enzymatic activity was quenched with equal volumes of Advanced DMEM F12 (Gibco, 12634028) and cells were pelleted at 300 xG for 5 min. For samples with in-well non-targeting control, BFP expression was swapped for mApple expression as previously described^23^. For extracellular markers (CD38, CD9, P2RY12), cells were incubated with antibodies for 30 min at 4 °C (FC block, Biolegend, 422302, 1:200; anti-CD38, R&D Systems, FAB2404P, 1:200; anti-CD9, Biolegend, 312104, 1:200; anti-P2RY12, Biolegend, 392108, 1:50).

For intracellular staining (IFIT1, IFITM3, IFI6, CXCL10, MX1, dsRNA) cells were fixed with eBioscience Intracellular Fixation and Permeabilization Buffer Set (Invitrogen, 888-8824-00) for 20 min at room temperature. After washing with permeabilization buffer, antibodies were added for 30 min at 4 °C in permeabilization buffer (anti-IFIT1, Cell Signaling, 20329S, 1:100; anti-IFITM3, Proteintech, 11714-1-AP, 1:100; anti-IFI6, Invitrogen, PA5-76860, 1:100; anti-CXCL10, Biolegend, 519504, 1:50; anti-MX1, CellSignaling, 37849S, 1:200; anti-dsRNA, Absolute Antibody, Ab00458-23.0, 1:250; anti-CD74, Biolegend 326808, 1:50; anti-CCL2, Biolegend, 505910, 1:50; anti-CCL13, R&D Systems, IC327G, 1:50; anti-TREM2, R&D Systems, MAB18281, 1:100). For dsRNA, anti-MX1, and anti-TREM2, cells were washed with Permeabilization Buffer before secondary antibodies in Goat anti-Rabbit 488 (Invitrogen, A-11008, 1:500) or Goat anti-Mouse 555 (Invitrogen, A-11029, 1:500) were added for 30 min.

Samples were analyzed on a BD LSR Fortessa X14 using BD FACSDiva software. Mean fluorescence intensity was calculated using FlowJo analysis software after gating for live, single cells. Data was collected from 3 independent differentiations with 3 replicate wells per experiment (n > 10,000 cells).

For stimulated conditions, IFNβ (Gibco, AF-300-02B) was added at 10 pM unless otherwise indicated 24 h before lifting. For analysis of secreted proteins (CXCL10) GolgiPlug (BD Biosciences, 555029, 1:1000) was added 6 hours prior to lifting. For analysis of dsRNA, cells were treated with 1 μg/mL of RNase A (EN0531) in low salt for 30 min at 37 °C. This occurred after fixation and permeabilization but before antibody staining.

### Immunocytochemistry

After washing with DPBS, cultures were fixed for 7 min with 4% paraformaldehyde before blocking for 1 hr in DPBS 0.01% Triton X-100, 5% goat serum. Antibodies were incubated in blocking buffer overnight at 4 °C (anti-MX1, Cell Signaling, 37849, 1:500; anti-IFITM3, Proteintech, 11714-1-AP, 1:500). Cultures are washed 3x with DBPS before adding secondary antibodies 1:1000 and Hoechst 1:10000 diluted in DPBS. After 30 min, cultures were washed 3x with DPBS and stored in 0.5% NaN_3_.Images were collected on the Molecular Devices Image Express Confocal HT.ai or IN Cell Analyzer 6000. Images were analyzed using CellProfiler.

### Lentivirus generation

Lentivirus was generated as described^62^. Briefly, HEK-293T were plated to achieve 80-95% confluence after 24 h. For 2 mL of media, 1 μg each of transfer plasmid and third generation packaging mix were mixed with 100 μL Optimem (Gibco, 31985088) and 12 μL TransIT-Lenti Transfection Reagent (Mirusbio, MIR 6604). Transfection mix was incubated for 10 min at room temperature before adding to HEK-293T cells. After 48 h, media was collected and filtered through a 0.45 μm PVDF filter. Lentivirus Precipitation solution (Alstem, VC125) was used per manufacturer’s protocols.

iPSCs were infected with virus as single cells at time of passage and allowed 48 h for expression before selection with 1 μg/mL puromycin for two passages or until >95% BFP positive populations were achieved. For CRISPR screening libraries, cells were transduced at an MOI of 0.3 to avoid double sgRNA transduction within the same cell.

### Genome-wide CRISPRi screen

For the genome-wide screen, seven sublibraries (CRISPRi_v2, “top5” sgRNAs) were cloned into as described previously^63^. For each sublibrary, iPSCs were transduced at an MOI of 0.3 with 1000x guide coverage (expression of each guide in at least 1000 cells). For all maintenance of iPSCs with CRISPRi libraries including routine passaging, freezing, and differentiation, cells were maintained with at least 1000x coverage ( > 1000 cells per sgRNA).

For IFIT1 screening, each sublibrary of iPSCs was plated to eight 15 cm plates at 10 million iPSCs per plate and differentiated as described above with TMP to induce CRISPRi activity starting at d0. On day 8 of differentiation, iPS-Microglia were treated with 10 pM IFNβ for 24 h. On day 9, iPS-Microglia were lifted with TrypLE for 10 min at 37 °C and quenched with TBS + 3% BSA + 0.5 µM EDTA. After lifting, cells were stained with Zombie Red Fixable Viability kit (Biolegend, 423110, 1:200) for 10 min at 4 C. Because formaldehyde-based fixation interrupts subsequent genomic DNA isolation and PCR, iPS-Microglia were fixed with zinc fixation buffer as previously described^64^ (0.1 M Tris-HCl, pH 6.5, 0.5% ZnCl_2_, 0.5% Zn Acetate, 0.05% CaCl_2_) overnight at 4 °C. Following fixation, cells were stained with IFIT1 in TBS + 3% BSA + 0.5 µM EDTA for 30 min at 4 °C. Antibody was washed off in TBS + 3% BSA + 0.5 µM EDTA and cells were strained through a 20 µm filter to remove clumps. For each library ≥80 M iPS-Microglia were sorted using BD FACSAria Fusion flow cytometer to ensure >1000x coverage of guides in both high and low IFIT1 populations. Cells were gated on live, single cells, and sorted into top 30% and bottom 30% of IFIT1 expression. After sorting, genomic DNA was isolated with NucleoSpin Blood L kit (Macherey-Nagel, 740954.20). sgRNA cassettes were isolated and amplified as previously described^62^ and sequenced on Illumina HiSeq-4000.

### CRISPRi screen analysis

CRISPRi screening libraries were analyzed as previously described^65^. Raw sequencing results were mapped with ‘sgcount’^65^ and ‘crispr_screen’^65^ was used to perform differential expression analysis using a ‘drop first’ weighting strategy and Benjamini-Hochberg multiple hypothesis correction. Significant guide enrichment was calculated for each sublibrary independently based on the non-targeting guides within that library. To control for variance from each gene being targeted by 5 independent single-guide RNAs, non-targeting control guides were grouped into ‘amalgam genes’ consisting of a random sampling from the group of all non-targeting guides. We implemented a z score cutoff of 5 to remove non-targeting controls with likely off-targeted effects. For display of all seven sublibraries^63^ within the same plot, log2(fold change) enrichment of guide frequencies in IFIT1-high vs IFIT1-low samples were normalized to the standard deviation of non-targeting guides for that sublibrary.

To parse known screen hits, Google Gemini 2.5 pro was used to generate a prompt for Gemini 2.5 deep research. Full prompt is provided in Supplementary Data 5. In brief, Gemini was prompted to determine the strength of information linking each gene hit to interferon signaling by grouping into one of three categories.

Category 1: Canonical interferon signaling genes that directly impact the signal transduction downstream of type I interferon. Typically includes genes well-documented in the canonical JAK-STAT pathway, such as JAK kinases, STAT transcription factors, IRFs, and other well-recognized type I interferon signaling components (e.g., TYK2, ISGF3, etc.).

Category 2: Genes with any paper or limited evidence suggesting they may impact interferon signaling, viral response, or nucleic acid sensing. This includes genes where there is some experimental or in silico evidence linking them to interferon signaling or antiviral pathways, but where the evidence is either indirect, limited in scope, or not as widely confirmed. Category 3: Genes with no published impact on interferon signaling. No significant literature, gene ontology annotation, or experimental evidence that directly ties them to type I interferon signaling or antiviral mechanisms.

### Reverse-transcriptase quantitative polymerase chain reaction

For independent validation of screening results, individual sgRNAs were added to iPSCs as described above in lentivirus production. sgRNA sequences available in Supplementary Data 8.

To assess the level of RNA knockdown for independent lines, RNA was extracted using the Quick-RNA Microprep Kit (Zymo, R1050). cDNA was generated using SensiFAST cDNA Synthesis Kit (Meridian Bioscience, BIO-65053). For quantitative PCR, SensiFAST SYBR Lo-ROX 2X Master Mix (Bioline; BIO-94005) was used with custom qPCR primers (Supplementary Data 8) from Integrated DNA Technologies. Amplification was quantified using the Applied Biosystems QuantStudio 6 Pro Real-Time PCR System using QuantStudio Real Time PCR software (v.1.3). Expression fold changes were calculated using the ∆∆Ct method, normalizing to housekeeping gene *GAPDH*.

### RNA-sequencing

iPS-Microglia were treated with 10 pM or 100 pM IFNβ on day 8 of differentiation. On day 9, iPSCs were lifted with TrypLE for 10 min at 37 °C. RNA was isolated with Quick-RNA Miniprep Kit (Zymo, R1055). For determination of IFNβ responsive genes, QuantSeq 3’ mRNA-Seq Library Prep Kit for Illumina FWD (Lexogen, 015UG009V0252) was used to prepare RNA-sequencing libraries per the manufacturer’s instructions. Library amplification was performed with 11 PCR cycles. For analysis of differentiation expression after CNOT10 knockdown, PIP-seq 3’ Single-Cell RNA kit v3.0 was used per manufacturer’s instructions. Libraries were sequenced on Illumina NextSeq-2000.

Transcripts were aligned to the human reference genome (GRCh38, release 42) using Salmon v.1.4.Transcripts were mapped to genes and quantified using tximeta.

Cells were filtered to only include those with a minimum count of 2,500. 5,000 of the most highly variable genes were selected with a minimum shared count of 10. Cells were then normalized to a target sum of 10,000 and log transformed. The highly variable genes were used to perform principal component analysis. Next, nearest neighbors were calculated using the top 15 principal components, selecting the 15 nearest neighbors. All single-cell filtering, transformation, and dimensionality reduction was conducted using ‘scanpy’^66^.

To measure perturbation effects in cells, we first calculated a perturbation signature for each cell by searching for the 20 nearest neighbors from non-targeting cells, and then removing technical variation so that the perturbation-specific effect can be revealed. This was done using the CalcPerturbSig() function from the ‘Mixscale’ developed by the Satija lab^67^ and results were stored in the PRTB assay in ‘Seurat’^68,69^. We then conducted differential expression analysis between CNOT10 knockdown (KD) and non-targeting control cells using a Wilcoxon rank-sum test, using the ‘presto’ package^70^ (https://github.com/immunogenomics/presto) as used in Seurat v5 but implemented via a custom version of the ‘Mixscale’ package (https://github.com/reetm09/mixscale)^42^. Our implementation modified the original approach in identifying differentially expressed genes (DEGs) by applying Benjamini-Hochberg correction for multiple hypothesis testing instead of Bonferroni correction. DEGs with a P value < 0.05 after adjustment were selected as significant DEGs.

We used the top 500 significant DEGs to quantify the magnitude of expression shift of each cell from non-targeting control cells. This was done by building perturbation vectors for an equal number of cells in *CNOT10* KD and control, where the length of each vector was equal to the number of identified DE genes, with expression values from the PRTB assay. By calculating the mean difference in perturbation vectors between *CNOT10* KD and control cells, we projected each cells’ perturbation vector onto the difference vector to obtain perturbation scores. Scores were standardized by calculating the mean and standard deviation from control cells. These standardized Mixscale scores were then used to weight cells in a negative binomial regression DE analysis, using the Run_wmvRegDE() function, such that cells with a stronger perturbation response had a greater contribution in the identification of DEGs. This final list of DEGs was then used for downstream analyses including volcano plots and functional interpretation.

## Supplementary information

## Data Availability

All processed data is included in supplemental tables for this manuscript. Genome-wide CRISPR screening data is also available through CRISPRbrain.org.
