# Supplementary material for: Regulators of interferon-responsive microglia uncovered by Genome-wide CRISPRi screening
Source: NPJ Dement. 2026 Sep 2;2(1):75. doi: 10.1038/s44400-026-00125-4 (PMC13538059; doi:10.1038/s44400-026-00125-4)
Supplement: Supplementary file 1 [file 44400_2026_125_MOESM1_ESM.pdf]

Supplementary Information for

Regulators of Interferon-Responsive Microglia Uncovered by Genome-wide CRISPRi Screening

Amanda McQuade, Vincent Cele Castillo, Venus Hagan, Weiwei Liang, Thomas Ta, Reet Mishra, Olivia Teter, Layla Gomes, Bianca Gonzalez, Noam Teyssier, Kun Leng, Martin Kampmann

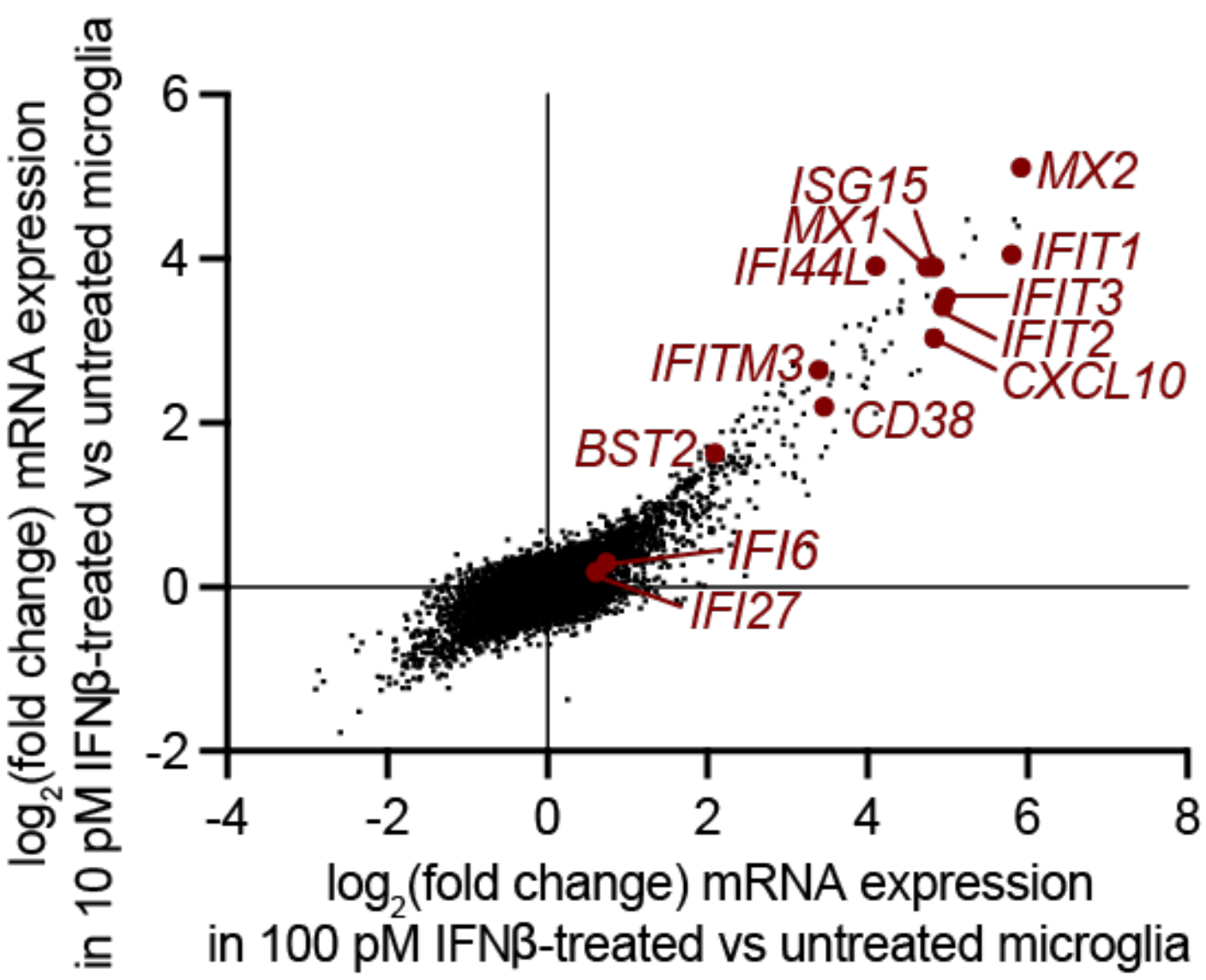

Supplementary Fig. 1. Comparison of transcriptional response of iPS-Microglia to 10 pM or 100 pM IFNβ. Differential expression of mRNA in 100 pM IFNβ-treated iPS-Microglia versus untreated are displayed on the x-axis. Differential expression of mRNA in in 10 pM IFNβ-treated iPS-Microglia versus untreated are displayed on the y-axis. Conserved ISGs from Fig. 1A are denoted in dark red. See also Supplementary Data 1.

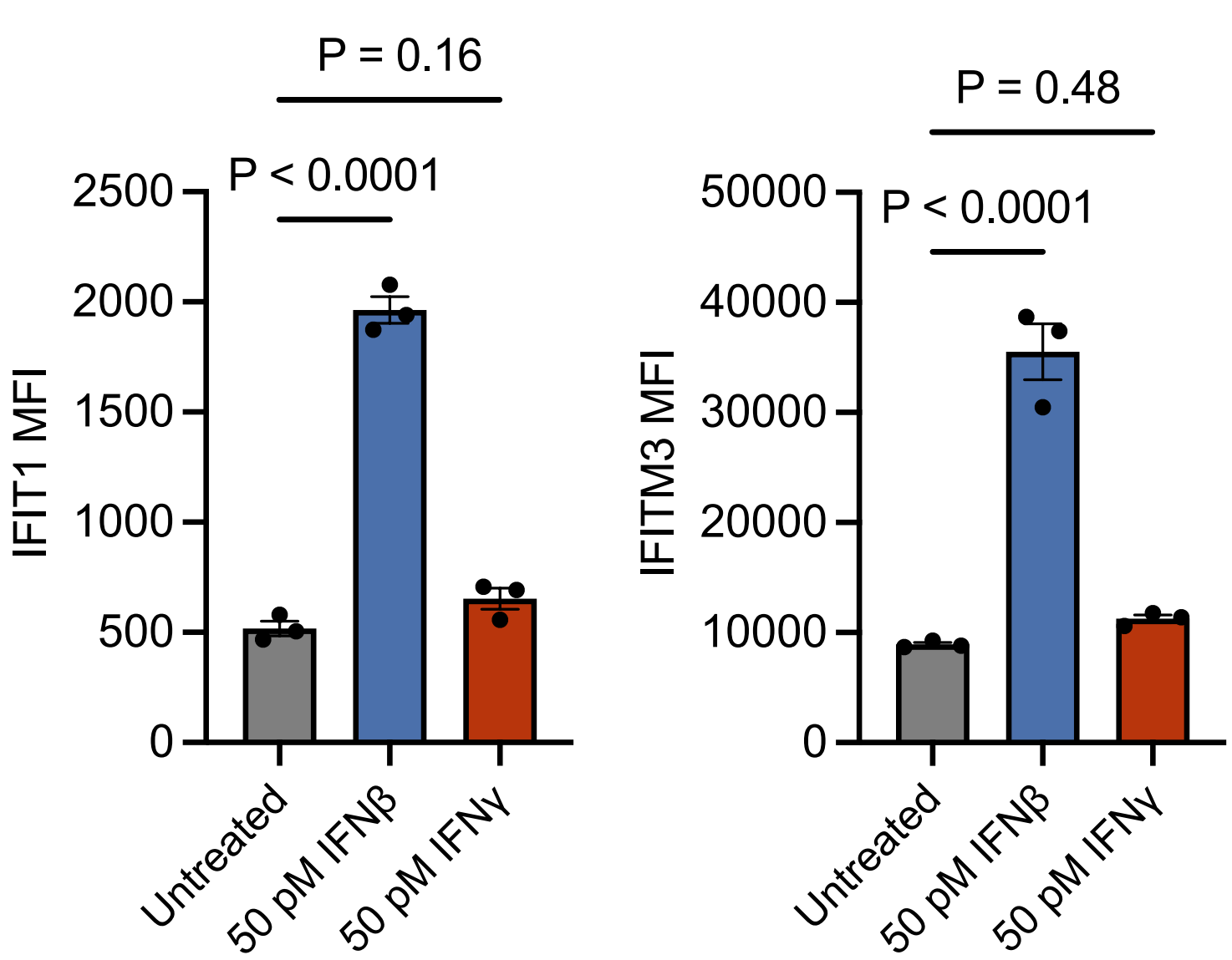

Supplementary Fig. 2. IFIT1 and IFITM3 specifically respond to type I interferon over type II. Median fluorescence intensity (MFI) of IFIT1 (left) or IFITM3 (right) after 24 hr treatment with 50 pM IFNβ or IFNγ. n=3 independent wells, > 10,000 cells per replicate. Bars represent mean +/- standard error, ANOVA, Tukey's multiple comparison test.

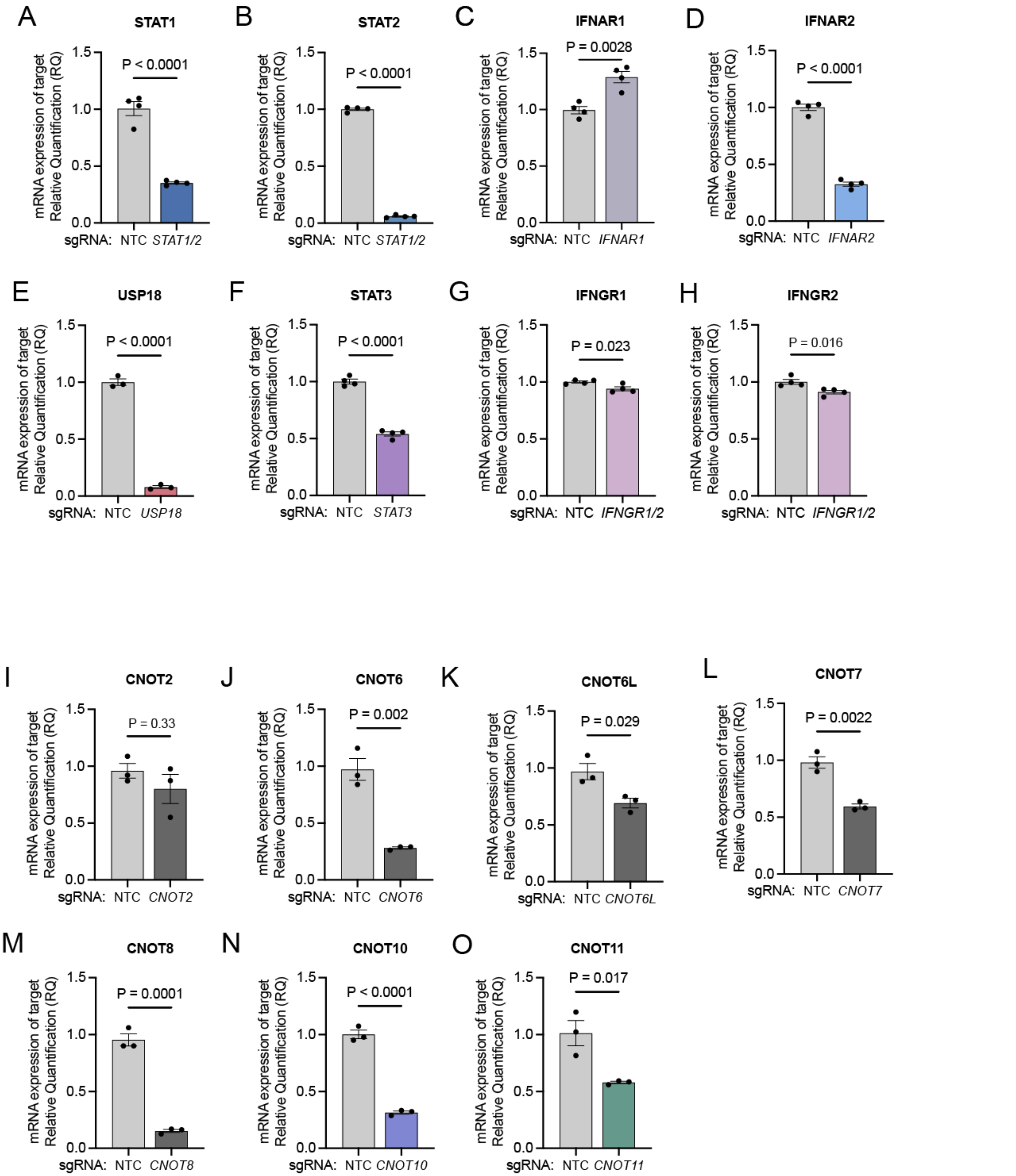

Supplementary Fig. 3. mRNA knockdown in individual knockdown lines. Quantitative reverse transcriptase polymerase chain reaction (RT-qPCR) was performed for each individual knockdown line compared to a non-targeting control sgRNA. n=3-4 independent wells, each point is an average of 3 technical replicates (3 wells for qPCR analysis). Bars show mean normalized to the non-targeting control +/- standard error, T-test.

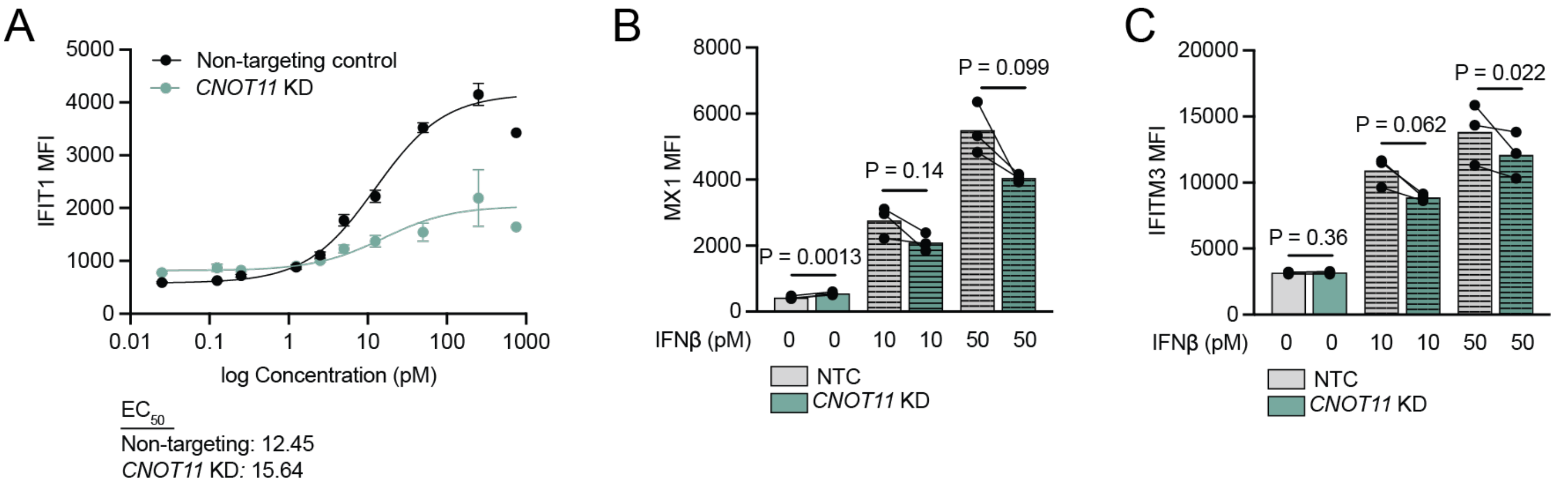

Supplementary Fig. 4. Knockdown of CNOT11 reduces levels of IFIT1 and IFITM3. (A) Dose response curves for interferon response in *CNOT11* knockdown (teal) vs non-targeting control (grey). Median fluorescence intensity (MFI) of IFIT1 was measured after 24 hours of treatment with IFNβ at the indicated concentrations. Data represent mean +/- standard error. n=3 independent wells > 10,000 cells per well. EC_{50} calculated as nonlinear fit [agonist] vs response in Prism. (B, C) Median fluorescence intensity (MFI) of (B) MX1 and (C) IFITM3 protein levels in *CNOT11* knockdown (teal) verses in-well, non-targeting control (grey) sgRNA. IFNβ was added at the indicated concentration for 24 hrs prior flow cytometry analysis. n=3 independent wells, > 10,000 cells per replicate. Bars represent mean +/- standard error, ANOVA, Tukey's multiple comparison test.

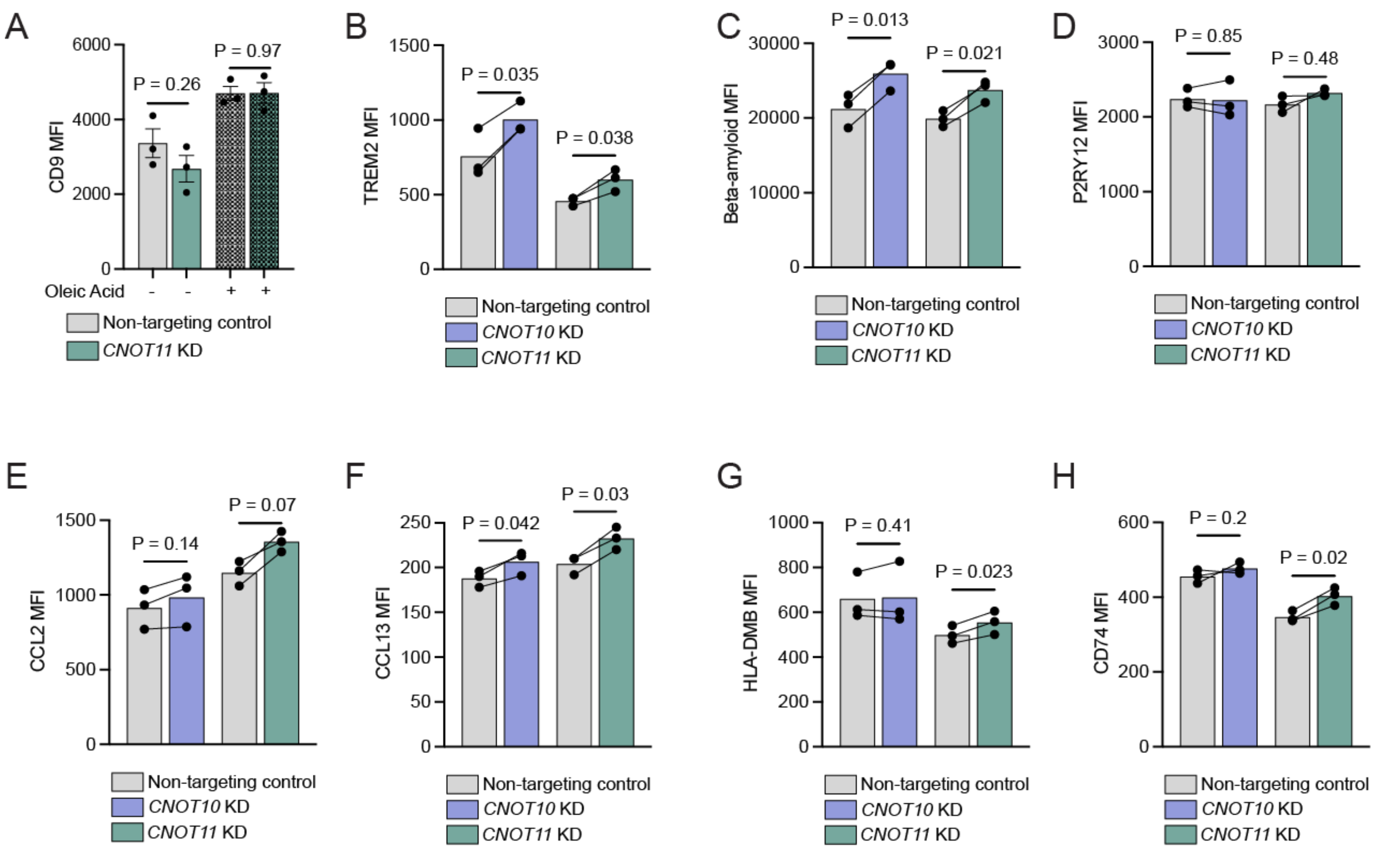

Supplementary Fig. 5. Knockdown of CNOT10 or CNOT11 does not inhibit other microglia activation states. (A) Median fluorescence intensity (MFI) CD9 surface protein levels in *CNOT11* knockdown (teal) versus non-targeting control (grey). 250 nM Oleic acid was added 24 hrs prior to flow cytometry analysis for the bars indicated. N=3 independent wells, > 10,000 cells per replicate. Bars represent mean +/- standard error, ANOVA, Tukey's multiple comparison test. (B) Median fluorescence intensity (MFI) TREM2 surface protein levels in *CNOT10* KD (periwinkle) or *CNOT11* KD (teal) microglia compared to in-well non-targeting control cells (grey) distinguished by nuclear fluorescent proteins. Each point represents an independent well, n > 10,000 cells analyzed per well. P values from paired Student's T-test. (C) Median fluorescence intensity (MFI) of beta-amyloid taken up by *CNOT10* KD (periwinkle) or *CNOT11* KD (teal) microglia compared to in-well non-targeting control cells (grey) distinguished by nuclear fluorescent proteins. Cells were exposed to 2 µg/mL fluorescent fibrillar beta-amyloid for 24 hrs. Each point represents an independent well, n > 10,000 cells analyzed per well. P values from paired Student's T-test. (D,H) Median fluorescence intensity (MFI) of activation state markers for (D) Homeostatic: P2RY12, (E,F) Chemokine-secreting: CCL2,CCL13, (G-H) Antigen-presenting (HLA-DMB, CD74) states. MFI displayed for *CNOT10* KD (periwinkle) or *CNOT11* KD (teal) microglia compared to in-well non-targeting control cells (grey) distinguished by nuclear fluorescent proteins. Each point represents an independent well, n > 10,000 cells analyzed per well. P values from paired Student's T-test.

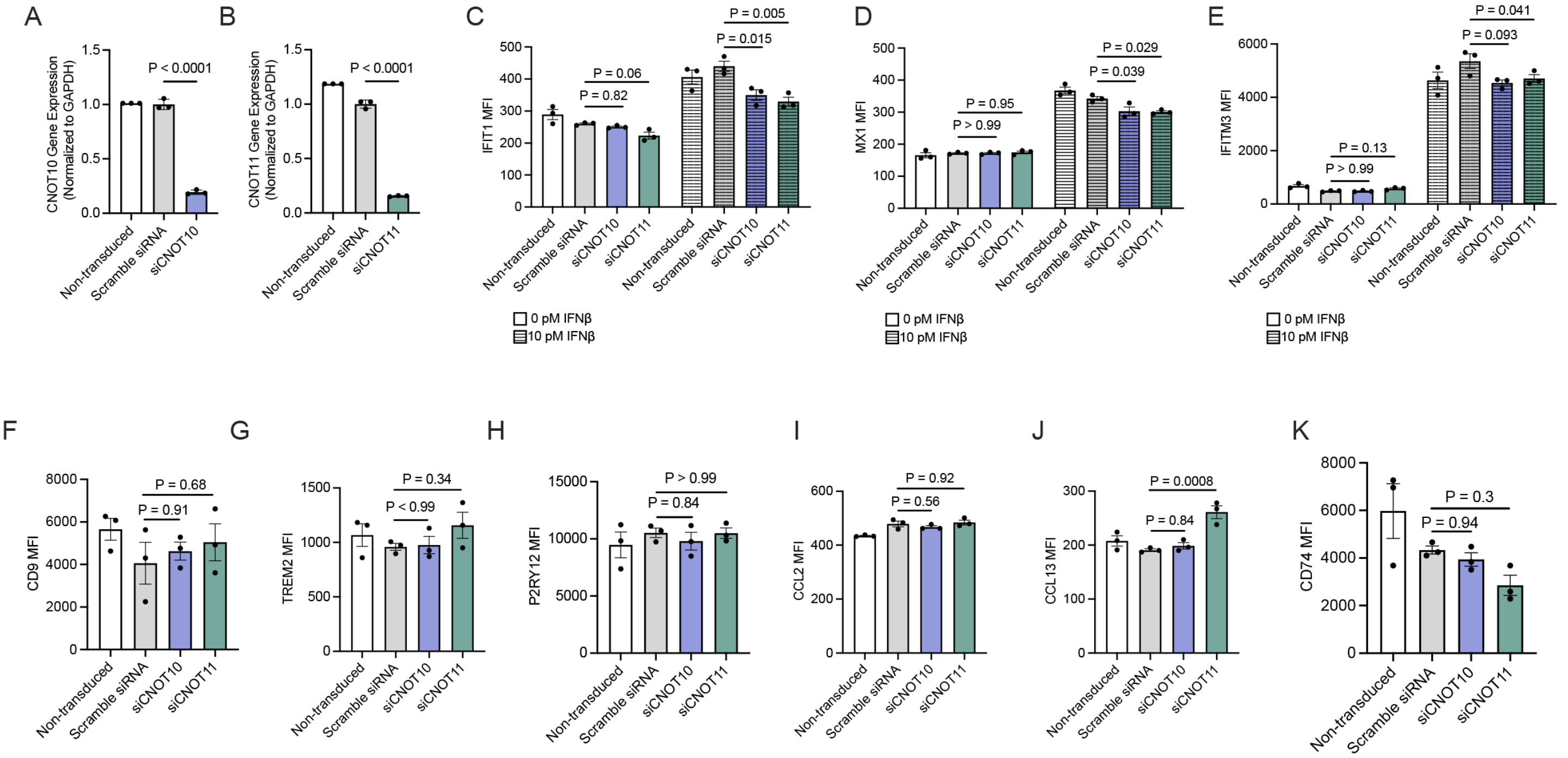

Supplementary Fig. 6. Confirmation of key phenotypes using RNAi in a separate iPSC background. Quantitative reverse transcriptase polymerase chain reaction (RT-qPCR) was performed for (A) *CNOT10* or (B) *CNOT11* for iPS-Microglia that were not transduced (white), transduced with a control siRNA (grey), or specific siRNA targeting *CNOT10* (periwinkle) or *CNOT11* (teal). n=3 wells, each point is an average of 3 technical replicates (3 wells for qPCR analysis). Bars show mean normalized to the non-targeting control +/- standard error, T-test. (C-H) Median fluorescence intensity (MFI) of activation state markers for (C,D, E) Interferon-response: IFIT1, MX1, IFITM3, (E,G) Disease-associated: (CD9, TREM2), (H) Homeostatic: P2RY12, (I, J) Chemokine-secreting: CCL2, CCL13, (K) Antigen-presenting: CD74. MFI displayed for Non-transduced (white), *CNOT10* KD (periwinkle), or *CNOT11* KD (teal) microglia compared to scramble siRNA cells (grey). Each point represents an independent well, n > 10,000 cells analyzed per well. P values from paired One-way ANOVA with Tukey multiple comparisons test.

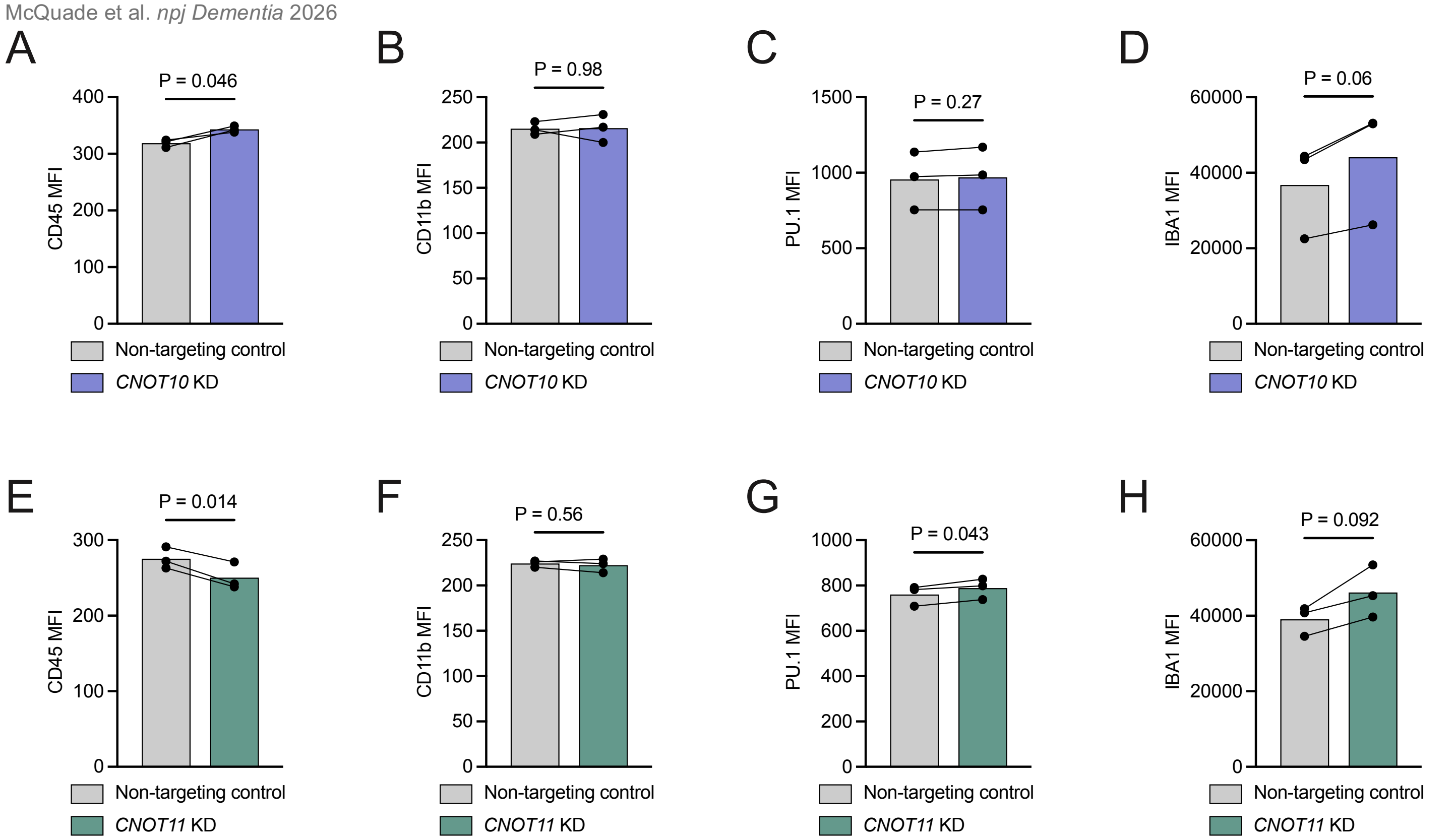

Supplementary Fig. 7. Knockdown of CNOT10 or CNOT11 does not inhibit other microglia activation states. Median fluorescence intensity (MFI) of microglia identity markers in *CNOT10* KD (periwinkle) or *CNOT11* KD (teal) microglia compared to in-well non-targeting control cells (grey) distinguished by nuclear fluorescent proteins. Each point represents an independent well, n > 10,000 cells analyzed per well. P values from paired Student's T-test.

Supplementary Data

Supplementary_Data_1_RNAseqIFNb.xlsx

Differential expression of iPSC-microglia treated with IFNβ. First tab represents 0 pM vs 100 pM IFNβ. Second tab represents 0 pM vs 10 pM IFNβ. Columns include the gene identifier, base mean expression across all samples, log2 fold change in untreated vs treated, P value and adjusted P value.

Supplementary_Data_2_IRMScreenGenomeWide.xlsx

Gene level results for genome-wide screen.

Supplementary_Data_3_GSEA.xlsx

Gene set enrichment analysis performed on all hits from the genome-wide screen using IDEA.

Supplementary_Data_4_GoogleGemini_Classification.xlsx

Output of Google Gemini analysis.

Supplementary_Data_5_GoogleGemini_Prompt

Task: You will receive a list of genes below. For each gene in this list, determine how strongly it is associated with type I interferon signaling (especially canonical interferon signal transduction) and/or antiviral response, and then classify it into one of the following three categories:
Category 1: Canonical interferon signaling genes that directly impact the signal transduction downstream of type I interferon. Typically includes genes well-documented in the canonical JAK-STAT pathway, such as JAK kinases, STAT transcription factors, IRFs, and other well-recognized type I interferon signaling components (e.g., TYK2, ISGF3, etc.).
Category 2: Genes with any paper or limited evidence suggesting they may impact interferon signaling, viral response, or nucleic acid sensing. This includes genes where there is some experimental or in silico evidence linking them to interferon signaling or antiviral pathways, but where the evidence is either indirect, limited in scope, or not as widely confirmed.
Category 3: Genes with no published impact on interferon signaling. No significant literature, gene ontology annotation, or experimental evidence that directly ties them to type I interferon signaling or antiviral mechanisms.

Instructions for Classification:
<u>Literature Review:</u> Search for each gene's name, aliases, and standard Gene Symbols in major literature databases (e.g., PubMed, Google Scholar, or references contained in curated resources).
Check if it is mentioned in the context of type I interferon, JAK-STAT signaling, IRFs, ISGs, antiviral immunity, or viral response research articles.
Take note of the quality and quantity of evidence: Is it cited in well-known reviews or studies explicitly as part of the core IFN pathway?
Is there only a single or a small handful of studies linking it tangentially to interferon or antiviral response?
What model systems are employed: please note the use of cell models, vertebrate models or other model organisms, human data, etc.

<u>Database & Ontology Queries:</u> Use gene ontology (GO) resources, such as the Gene Ontology website, and official pathway repositories (e.g., KEGG, Reactome, or WikiPathways) to check for terms like: "type I interferon signaling pathway," "response to virus," "immune response," "JAK-STAT cascade," etc. If the gene is annotated with GO terms clearly indicating involvement in "type I interferon signaling pathway," "viral defense," or "immune response," prioritize that information.

<u>Categorization Based on Evidence:</u>
Category 1 (Canonical IFN signaling): The gene is frequently cited and recognized as a core component of type I IFN signaling (e.g., direct effectors in the JAK-STAT cascade, known IRFs, or well-characterized interferon-stimulated genes).

Category 2 (Few studies or indirect involvement): The gene appears in at least a few publications linking it to interferon pathways or antiviral response, but it is not widely recognized or directly annotated as a canonical signaling component.

May be implicated in modulating IFN pathways or viral replication in cell-based or organismal studies, but evidence is not robust or is limited to specialized contexts.

Category 3 (No known impact):The gene has no significant mention in the context of interferon biology or viral response in literature or gene ontology/pathway databases.

Report Format- Return a table or structured data containing:
Gene Symbol: The official Gene Symbol (e.g., as designated by HGNC).
Common Aliases: Any synonyms or alternative names.
Category (1, 2, or 3).
Evidence Summary: (Optional but recommended) A short note with either a citation or a brief description of the supporting rationale (for categories 1 and 2), or a statement that no evidence was found (for category 3).
Model System: For genes classified in Category 2, please additionally report whether the existing evidence comes from experiments performed in cell models, human systems, or other model organisms (please define several categories and specify the model system in an additional column).

Important Notes:

1. If conflicting data emerges, prioritize recent and well-reviewed publications or authoritative databases.
2. If a gene is widely known to be essential in another closely related pathway (e.g., NF-κB activation) but only loosely tied to IFN signaling, consider whether it truly belongs in Category 2 or is best assigned Category 3.
3. Remember: The objective is to comprehensively classify each of these genes into exactly one category based on the current state of scientific evidence.

Please ignore the paper 'Regulators of Interferon-Responsive Microglia Uncovered by Genome-wide CRISPRi Screening'

Supplementary_Data_6_GSEA_ClassIII.xlsx
Gene set enrichment analysis performed on Class III genes identified by Google Gemini using IDEA.

Supplementary_Data_7_CNOT10RNAseq.xlsx
Differential expression of CNOT10 KD microglia versus Non-targeting control cells.

Supplementary_Data_8_sgRNA_Sequences_and_primers.xlsx
Protospacer sequences for sgRNAs used in this study.
